# Bibliometric Evaluation of Global Tai Chi Research from 1980–2020

**DOI:** 10.3390/ijerph18116150

**Published:** 2021-06-07

**Authors:** Yanwei You, Leizi Min, Meihua Tang, Yuquan Chen, Xindong Ma

**Affiliations:** 1 Division of Sport Science & Physical Education, Tsinghua University, Beijing 100084, China; youyw20@mails.tsinghua.edu.cn (Y.Y.); mlz20@mails.tsinghua.edu.cn (L.M.); 2 School of Kinesiology, Shanghai University of Sport, Shanghai 200438, China; yunhua581@163.com; 3 Institute of Medical Information/Library, Chinese Academy of Medical Sciences and Peking Union Medical College, Beijing 100020, China; chen.yuquan@imicams.ac.cn

**Keywords:** Tai Chi, exercise and health science, bibliometric analysis, visualization analysis

## Abstract

While studies on the health benefits of Tai Chi have sprung up over the past four decades, few have engaged in collecting global data, estimating the developing trends, and conducting reviews from the perspective of visualization and bibliometric analysis. This study aimed to provide a summary of the global scientific outputs on Tai Chi research from 1980 to 2020, explore the frontiers, identify cooperation networks, track research trends and highlight emerging hotspots. Relevant publications were downloaded from the Web of Science Core Collection (WoSCC) database between 1980 and 2020. Bibliometric visualization and comparative analysis of authors, cited authors, journals, co-cited journals, institutions, countries, references, and keywords were systematically conducted using CiteSpace software. A total of 1078 publications satisfied the search criteria, and the trend of annual related publications was generally in an upward trend, although with some fluctuations. China (503) and Harvard University (74) were the most prolific country and institution, respectively. Most of the related researches were published in the journals with a focus on sport sciences, alternative medicine, geriatrics gerontology, and rehabilitation. Our results indicated that the current concerns and difficulties of Tai Chi research are “Intervention method”, “Targeted therapy”, “Applicable population”, “Risk factors”, and “Research quality”. The frontiers and promising domains of Tai Chi exercise in the health science field are preventions and rehabilitations of “Fall risk”, “Cardiorespiratory related disease”, “Stroke”, “Parkinson’s disease”, and “Depression”, which should receive more attention in the future.

## 1. Introduction

In traditional Chinese exercise and medicine, Tai Chi plays an extremely important role in health promotion and its positive effect has been widely reported [1,2,3,4,5,6]. As a traditional martial art widely practiced in China for centuries, Tai Chi is well known for its slow and graceful rhythm transformation. In December 2020, the United Nations Education Scientific and Cultural Organization (UNESCO) announced that Tai Chi would be included in the representative list of the intangible cultural heritage of humanity. Tai Chi, also known as Taiji, Tai Chi Quan or Tai Chi Chuan, was originated in China in the 18th century. In this research, we used the term Tai Chi to represent Tai Chi Chuan and its related activities. The definition of the term Tai Chi derived from both the Chinese Taoist and traditional Confucian philosophy [7]. A few hundred years ago, a famous martial artist in the late Ming Dynasty named Wangting Chen first invented and put forward systematically a Tai Chi theory system. Tai Chi was performed in a variety of forms, which were named after different Chinese families such as Chen, Yang, Wu, Sun, etc. The diverse styles shared many basic theories but differed in training methods such as posture, rhythm, and sequence of movements [8]. Among all types of Tai Chi, the most classic and widely spread was the Yang family-style Tai Chi formed by 24 postures [1]. Tai Chi exercise can be practiced in numerous places, in an office, at home, at school, etc. No additional equipment is required, and even four square meters of flat ground is enough. Considering that Tai Chi is an all-age applicable, low-cost, space-saving and more enjoyable strategy, it has gained favorable acceptance in both Eastern and Western regions as a promising low to medium intensity, mind-body exercise.

In the past four decades, a growing number of studies focusing on the health benefits of Tai Chi in various chronic diseases has been widely reported. Some systematic reviews have investigated the effectiveness on Tai Chi in a variety of problems and diseases, such as postural control and fall prevention [9,10,11,12], musculoskeletal disorders [13,14,15], cardiovascular and respiratory conditions [16,17,18,19], and psychological problems [20,21,22,23]. However, few have engaged in collecting global data, estimating the developing trends, and conducting reviews from the perspective of systematic visualization and bibliometric analysis. Therefore, it is necessary to conduct this study to review the past research status and update the latest frontier in Tai Chi research.

Bibliometrics is a quantitative analysis strategy which involves mathematics and statistics methods to calculate the overall trends of research activities and identify the connections between relevant authors and institutions [24,25,26]. Based on multiple indexes like journals, authors, countries and institutions, it can conduct an in-depth evaluations of research trends and the focus of scientific production [27,28,29]. The results provide guidance and advice about novel research tendencies, future directions and decision making. CiteSpace is a Java-based scientific mapping software developed by Chen (Drexel University, Philadelphia, PA, USA) for bibliometric and comparative analysis [30,31]. By presenting multitudinous data, it can provide emerging trends and transition patterns in the scientific literature.

To the best of our knowledge, this is the first study that applied bibliometric visualization tools into systematic investigation about the active authors, journals, countries, institutions, references, and keywords involved in global Tai Chi research. We also combined the quantitative and qualitative analysis to evaluate the progress and evolution of Tai Chi by using the CiteSpace V software. It is noteworthy that this research did not involve the elaboration of principles or methods of the specific movements in Tai Chi exercise, but attempted to demonstrate a visualization strategy to analyze developments and explore frontiers on Tai Chi research and its applications in health promotion over the past 40 years, identify cooperation networks, track research trends and highlight current hotspots in order to offer references for further research. We considered that this study can not only help researchers extract potential valuable information for deeper investigation, but also provide them with meaningful guidance in the selection of frontier topics.

Notably, this paper answered the following three key questions.

(i) Which countries, institutions, journals, authors, references have played important roles in global Tai Chi research?
(ii) What are the most promising domains that Tai Chi exercise can be applied to in health science in the future?
(iii) What are the existing concerns and difficulties in Tai Chi research?

## 2. Materials and Methods

**Figure 1:**
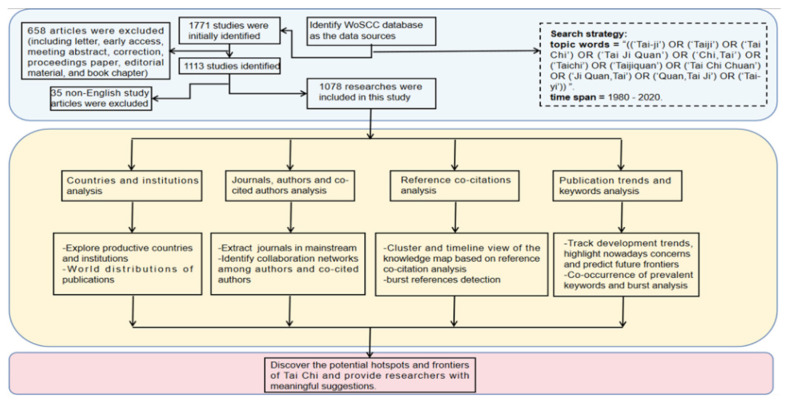
The overview and flowchart of the study design.

All studies were collected from Web of Science Core Collection (WoSCC) database on 10 January 2021. The reason for using WoSCC is that it contains comprehensive citation index records which include numerous influential and high-quality journals all over the world. Considering that Tai Chi is an interdisciplinary research, which involved in the fields of sports science, rehabilitation and complementary medicine, etc., so it is reasonable to use a comprehensive database rather than a database of a specific discipline like PubMed. In addition, we wanted to conduct a global study on Tai Chi, so China National Knowledge Infrastructure (CNKI) might not be an ideal choice. When it comes to Scopus, one of its weaknesses is that it does not have references before the year 1996 [32], and cannot provide a holistic retrieval in this condition. In terms of classification, WoS is also one step higher than Scopus in representing metadata lists. Actually, search results in WoS are not presented by author, year, subject and document types like Scopus, but also by institutions and countries [32], which is convenient for scholars to analyze the distribution of the research organizations. Moreover, WoSCC has been used as a data source in some previous bibliometric studies [33,34,35,36,37,38], which further proves its feasibility especially in the natural science domains. The study design was shown in Figure 1.

The search keywords entered into the WoSCC database were as follows: topic words = ((“Tai-ji”) OR (“Taiji”) OR (“Tai Chi”) OR (“Tai Ji Quan”) OR (“Chi,Tai”) OR (“Taichi”) OR (“Taijiquan”) OR (“Tai Chi Chuan”) OR (“Ji Quan,Tai”) OR (“Quan,Tai Ji”) OR (“Tai-yi”)), time span = 1980–2020. We further screened the included literature by title in order to ensure the relevance. All the subject-related records retrieved from the database were subsequently saved, sorted, and extracted. This research only selected articles or reviews for analysis and the language was limited to English. Other document types and non-English articles were excluded. Basic information for each study was converted to text documents, such as countries, institutions, publishing journals, authors, and references. A total of 1078 records finally matched the search criteria. We conducted the analysis by using the CiteSpace version V (Drexel University, Philadelphia, PA, USA) and Microsoft Excel 2016 software (Microsoft Corporation, Redmond, WA, USA). CiteSpace has been broadly recognized as an applicable bibliographic and comparative analysis tool. Visualization of knowledge domains, collaborative networks, reference citations were calculated, and then landmark literature, emerging trends, and hidden implications were detected. Parameters applied included publication amount, impact factor, centrality, and occurrence/citation burst. Impact factor (IF) is an international standard criteria for evaluating the impact of a journal [39] and the impact factor in this paper was on the basis of the Journal Citation Reports (2019). Centrality is an index for measuring the importance of a node in a network, and a node with large size typically indicates high occurrence or citation frequency as a pivotal point [31,40,41]. Occurrence burst represents a term that occurs frequently over a given period which can be considered as research hotspots [40,42,43]. In addition, we used the Microsoft Excel 2016 software to describe and predict the publication trend of Tai Chi articles. The function model was as follows: f(x) = ax^3^ + bx^2^ + cx + d, in which x represented for year of publication and f(x) demonstrated the cumulative amount of publications. In this way, we collected scientific publications on Tai Chi research in the last 40 years using bibliographic and visual analysis to discover the potential hotspots and frontiers of Tai Chi and aimed to provide researchers with meaningful suggestions.

## 3. Results

### 3.1. Countries and Institutions

We conducted an independent analysis of countries and institutions involved in Tai Chi research. Scholars from more than 40 territories and 450 institutions have contributed to this research field. There were 42 countries publishing Tai Chi research. The details of the top 10 countries and institutions were presented in Table 1. China took the lead and had the highest amount of publications (503), which accounted for almost half of the total number of published articles. The United States was the second most productive country, with 394 publications, followed by Australia (67), Canada (59), UK (49) and South Korea (47). The top six countries’ contributions were all above 40 articles respectively, which implied that they all made major contributions to the research achievements.

**Figure 2:**
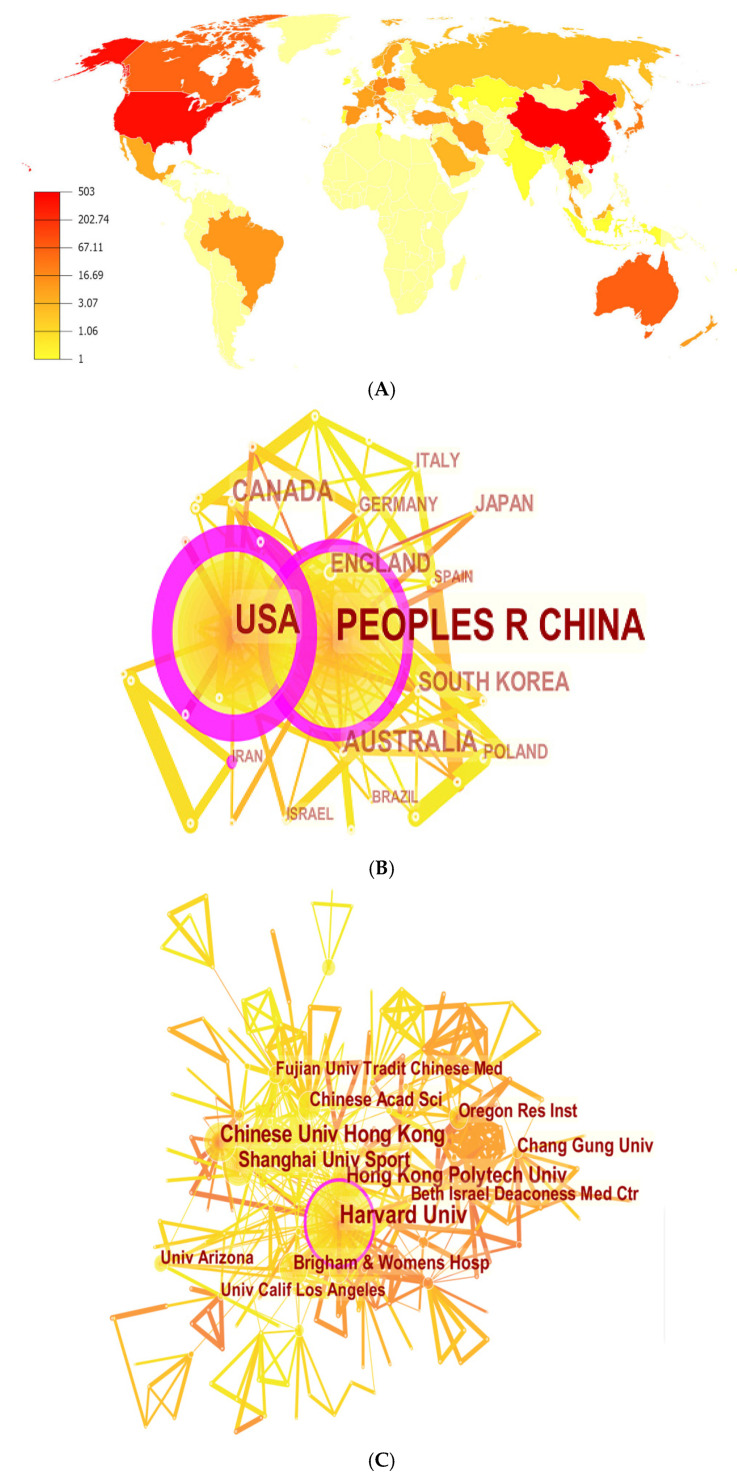
(**A**) Map of world distribution with publications in the field of Tai Chi research; (**B**) Map of countries with publications in the field of Tai Chi research; (**C**) Map of institutions with publications in the field of Tai Chi research.

The distribution of these countries is presented in Figure 2A, and the collaborations among them were generated by CiteSpace V with 42 nodes and 92 links and are presented in Figure 2B. When it comes to the centrality, a wider circle indicates higher centrality [44]. The United States had the highest centrality (0.66), followed by China (0.38), Australia (0.09) and Canada (0.08). Institutions participating in Tai Chi studies are displayed in Figure 2C. As listed in Table 1, universities accounted for more than half of the top 10 research institutions. Harvard University was the leading institution (74 publications), followed by Chinese University of Hong Kong (51 publications), the Hong Kong Polytechnic University (45 publications) and Shanghai University of Sport (39 publications). 

The network map of institution cooperation was generated using CiteSpace V and 459 nodes accompanied with 805 links were built simultaneously. In terms of centrality, these four universities mentioned above were first-tier institutions, Furthermore, six of the top 10 institutions belonged to China and four of which were subordinate to the United States, suggesting that these two countries occupied the dominant positions in the field of Tai Chi research.

### 3.2. Active Journals, Authors and Co-Cited Authors

Active journals included both citing and co-cited journals. All articles related to Tai Chi research were published in more than 450 citing journals and the number of journals which cited Tai Chi publications were over 730. The number of publications among the top 10 citing journals varied from 14 to 42 (average 22.2), made up nearly one fifth of the total number of publications. As shown in Table 2, *Evidence Based Complementary and Alternative Medicine* (IF 2019 = 1.813) published the highest number of articles (42 publications, 3.90%), followed by *Archives of Physical Medicine and Rehabilitation* (2.88%, IF 2019 = 3.098), *Complementary Therapies in Medicine* (2.32%, IF 2019 = 2.063), *American Journal of Chinese Medicine* (2.04%, IF 2019 = 3.682) and *Journal of the American Geriatrics Society* (1.86%, IF 2019 = 4.180). Among these top 10 journals which published the highest number of articles, the impact factors of them were between 1.552 to 5.200 (average of 2.865). Publishing Tai Chi works in a high impact factor journal still remained to be a challenging task.

**Figure 3:**
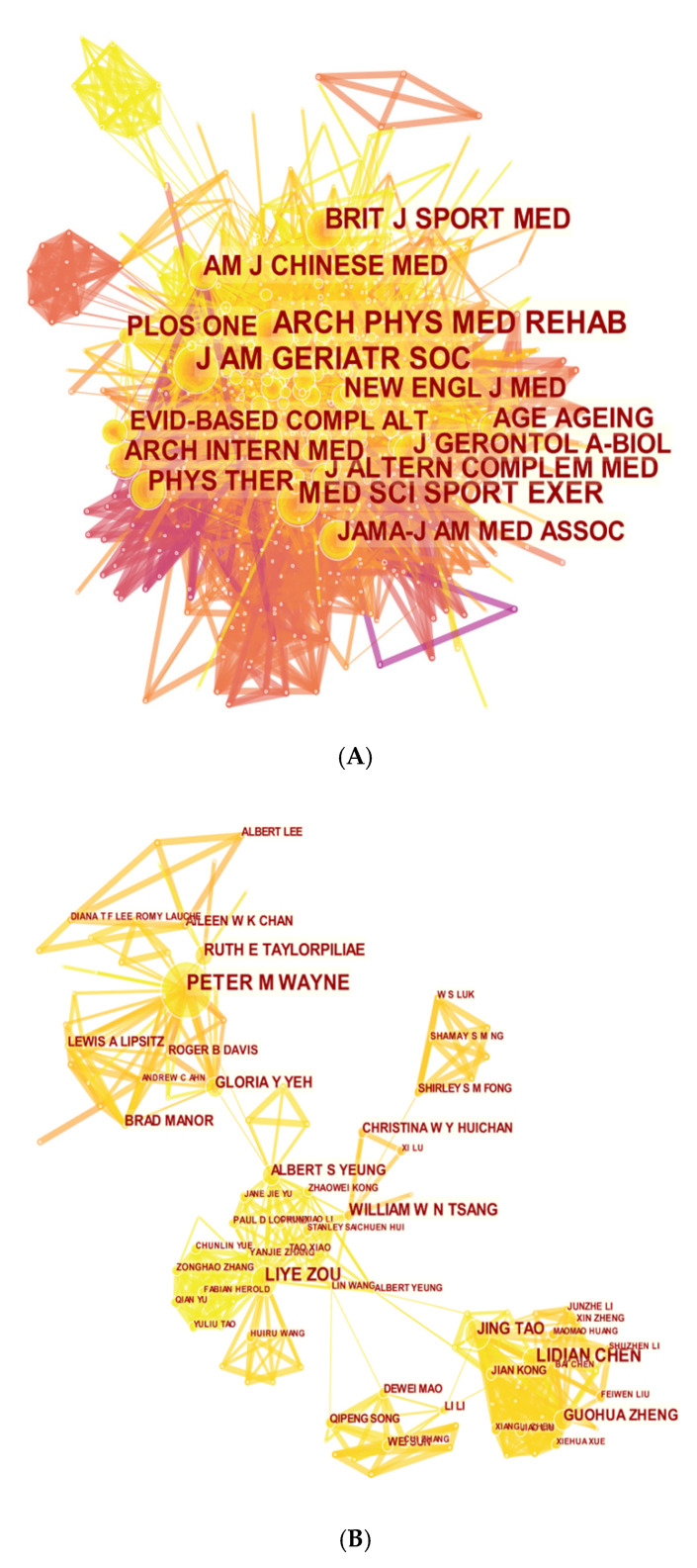

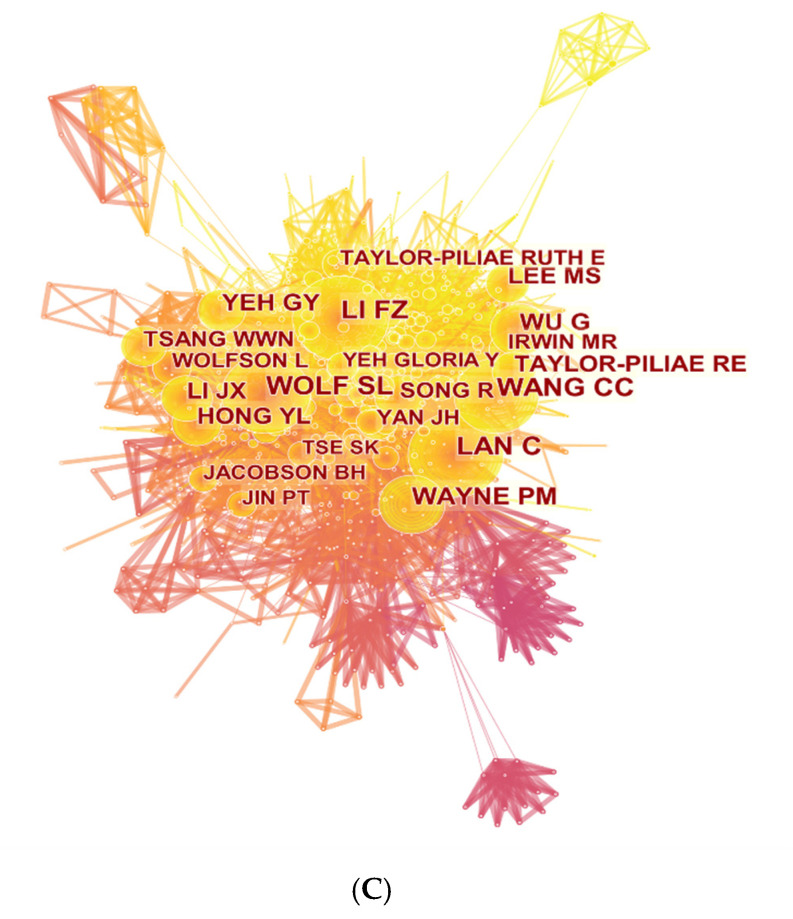
(**A**) Map of active journals with publications in the field of Tai Chi research; (**B**) Map of active authors with publications in the field of Tai Chi research; (**C**) Map of active co-cited authors with publications in the field of Tai Chi research.

In the co-cited journal list, *Journal of the American Geriatrics Society* was the number one journal with 599 counts. *Archives of Physical Medicine and Rehabilitation, Medicine and Science in Sports and Exercise* also contributed 534 and 424, respectively. The high co-citation counts showed that these journals had excellent quality and academic dominant in Tai Chi research. The co-citation journal network map was plotted by CiteSpace V with 745 nodes and 7169 links. As shown in Figure 3A, the diverse distribution of journals that published papers related to Tai Chi provided multiple choices for scholars to submit their works to. We can also find that most of the publications were focused on the categories including sport sciences, alternative medicine, geriatrics gerontology, and rehabilitation.

A total of 662 authors and 907 co-cited authors contributed to Tai Chi research. The top 10 scholars and co-cited scholars involved in the field of Tai Chi research were presented in Table 3. The top 10 authors published in total 168 studies, accounting for 12.35% of all published papers on Tai Chi research. Wayne took the lead with 32 publications, followed by Zou with 17 publications and Chen with 16 publications, respectively. The top five co-cited authors were Li, Lan, Wang, Wolf, and Wayne, and the number of citations of these authors’ all exceeded 200 counts. Table 3 shows the top 10 most co-cited references as well. These references could be considered as the most popular papers in the Tai Chi field. The top co-cited reference was authored by Li and was published in *New England Journal of Medicine* (the journal leader in clinical medicine, IF 2019 = 74.699).

Cooperations between authors and co-cited authors were analyzed for the identification of potential partnerships. Collaboration partnerships were represented by connections among nodes and the thickness of the connection represents the closeness of cooperation. The merged co-relationship of authors in Figure 3B was composed of 663 nodes and 1096 links. Figure 3C illustrates the connections between co-cited authors (nodes = 907, links = 6498). The knowledge map between authors and co-cited authors can not only provide numerous information on important research groups and potential partners, but also help researchers to identify and establish cooperations.

### 3.3. Reference Co-Citations

Reference co-citation analysis was one of the most important measurements in bibliometric research which usually used to identify research hotspots in a specific research field [45]. Table 3 showed the top 10 most co-cited references as well. These references could be considered as most popular research in the Tai Chi field. The top co-cited reference was authored by Li and was published in *New England Journal of Medicine* (the leading journal in clinical medicine, IF 2019 = 74.699).

**Figure 4:**
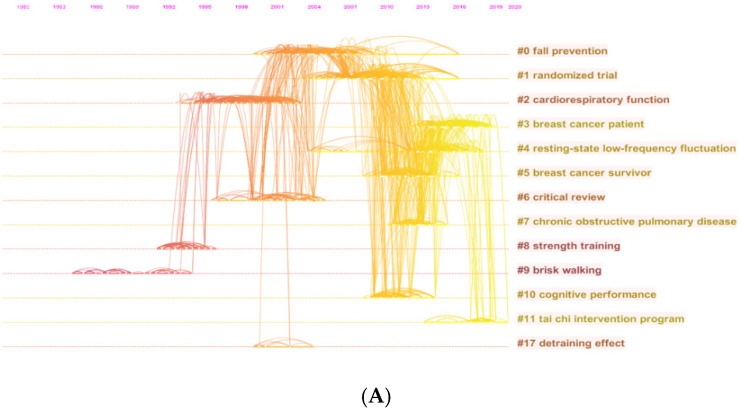

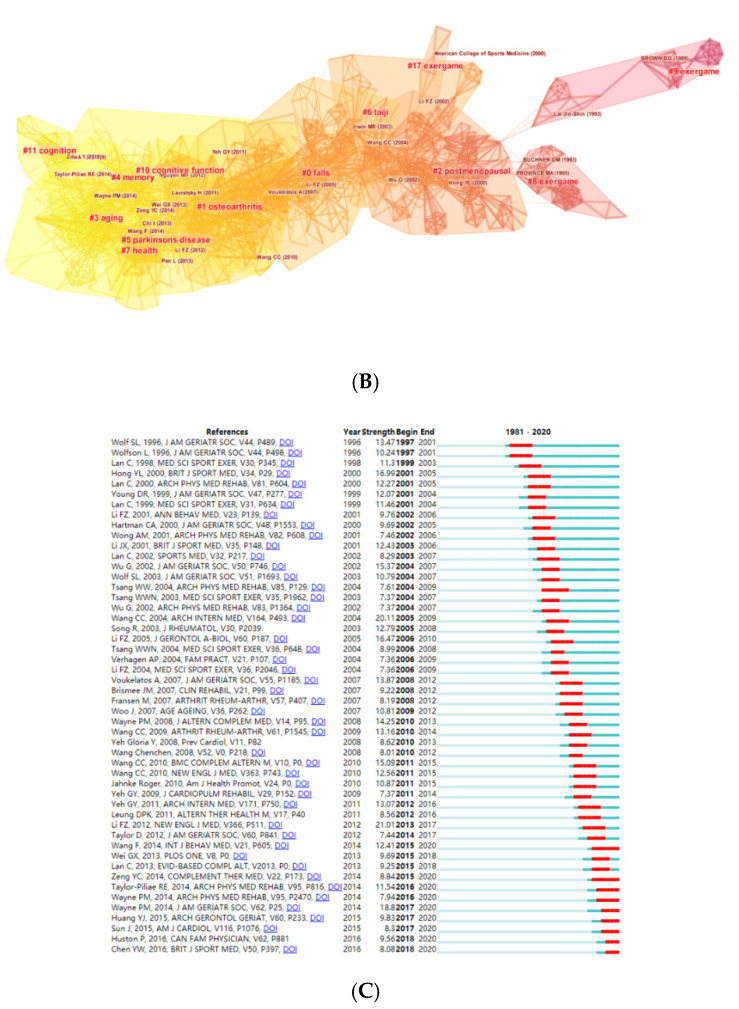
(**A**) Map of timeline view based on the reference co-citation analysis in the field of Tai Chi research; (**B**) Map of cluster view based on the reference co-citation analysis in the field of Tai Chi research; (**C**) Map of the top 50 burst references based on the reference co-citation analysis in the field of Tai Chi research.

Articles and their co-citation relevant data were retrieved and then major clusters were created. The timeline view for the clusters, which narrated the time interval and research advance in the progress and evolution of Tai Chi research was presented in Figure 4A. “Fall prevention” was the most significant cluster (#0) out of the 13 clusters, followed by cluster #1 (randomized trail) and cluster #2 (cardiorespiratory function). References with strongest citation burst were supposed as the research frontiers in the foreseeable future [46,47].

We analyzed reference co-citation conditions by constructing knowledge domains of clusters. As shown in Figure 4B, the articles associated to Tai Chi field between 1980 and 2020 were divided into 13 major research hotspots. Each cluster represented the citation index, achievement field and key literature series within a period of time. As presented in Figure 4C, the top 50 burst references in this study were examined and distinguished by the index of strongest citation bursts. There were nine articles had its citation bursts until 2020, which implied that Tai Chi research has attracted high attention in recent years.

### 3.4. Publication Trends and Keywords

**Figure 5:**
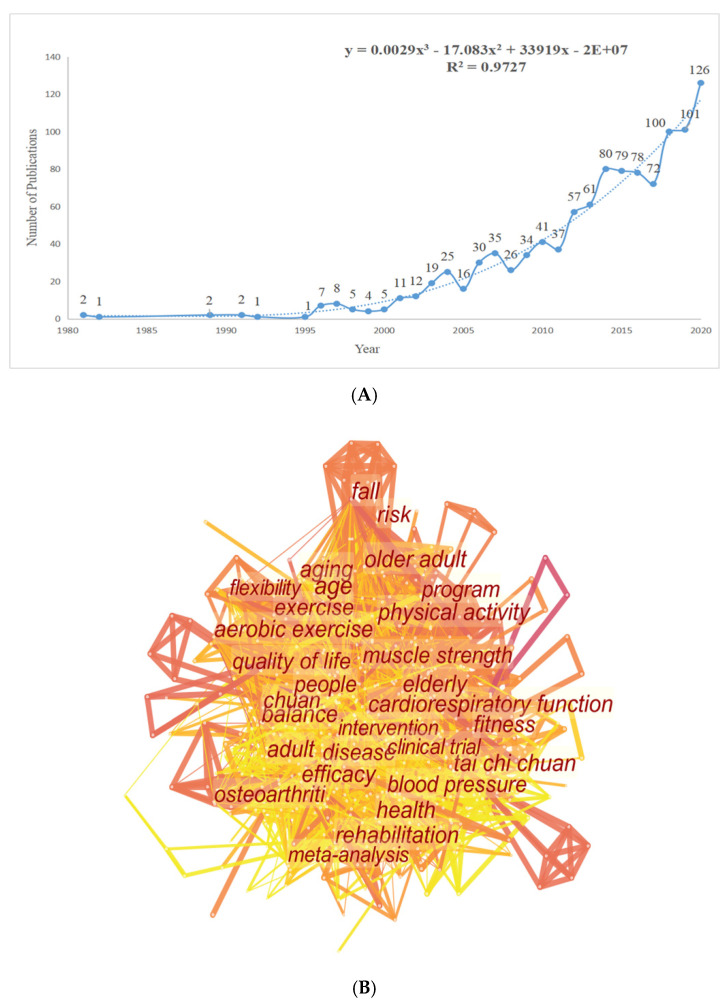

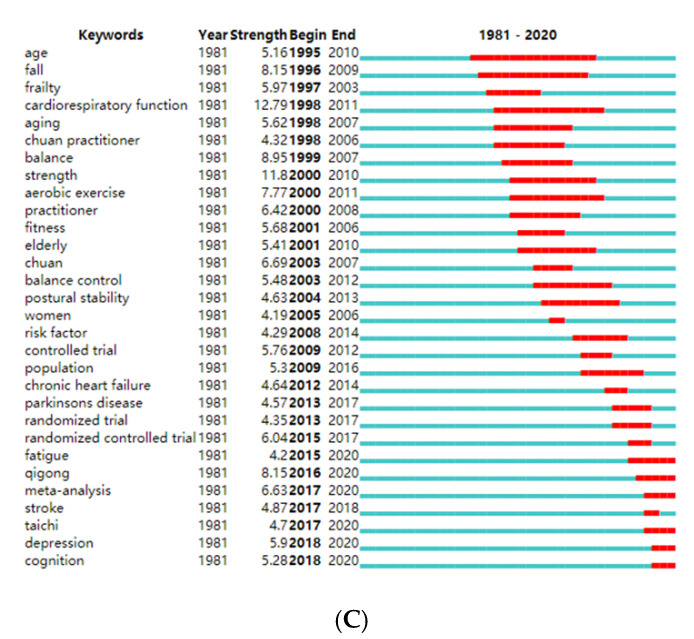
(**A**). Map of publication trends in the field of Tai Chi research; (**B**) Map of conspicuous keywords in the field of Tai Chi research; (**C**) Map of the top 30 burst keywords in the field of Tai Chi research.

Among the 1078 included literature, the relationship between the amount of articles per year and the development tendency on Tai Chi research were shown in Figure 5A. Quantitative analysis demonstrated that the overall trend of publications increased from two in 1981 to 126 in 2020. To be specific, few publications related to Tai Chi was included in the database before 1980. The effect of Tai Chi was unknown and underestimated before that time point. Actually, with the development of sports science and complementary medicine, the health benefits of Tai Chi gradually began to be recognized and explored. Since 1981, the number of Tai Chi-related articles included by WoSCC have gradually increased. In 2001, the number of annual publications reached 11, exceeding 10 for the first time, and then it maintained an overall increasing tendency although it experienced some fluctuations in the next 13 years. then it had reached 80 in 2014. After 2014, the number of papers related to Tai Chi experienced a leap again, and reached a peak in 2020, with 126 publications. The number of annual publications on Tai Chi has increased significantly within the last four decades and a growth trend model (R^2^ = 0.9727) indicated that Tai Chi and its applications have received increasing attention, and further research into these fields is still ongoing.

Several conspicuous keywords involved in Tai Chi research are presented in Figure 5B. The top 30 keywords with the strongest occurrence burst are shown in Figure 5C. An occurrence burst, which indicates a continuous increase in occurrence during a certain time interval, can reflect development in leading-edge topics in a certain research domain [40,42]. Between 1980 and 2020, the top five keywords which took the lead with the highest citation burst in Tai Chi research were “cardiorespiratory function” (12.79), “strength” (11.80), “balance” (8.95), “fall” (8.15), “qigong” (8.15). The applications of Tai Chi in health promotion contained the following five key domains: “Fall risk”, “Cardiorespiratory related disease”, “Stroke”, “Parkinson’s disease”, and “Depression”, which were worthy of attention and would be further discussed in the next part.

## 4. Discussion

Exercise is medicine. As an old Chinese proverb says “stagnant water is putrid”, which indicates that exercise plays a crucial role in maintaining body fitness and health, and thereby, longevity. Tai Chi, as an ancient wisdom on exercise and health maintenance method, has become one of the most favorable exercises and health promotion methods for all age groups in different countries and regions of the world.

From Figure 5A, we can observe that publications on Tai Chi can be divided into three stages: the initial stage (1980–2000), second stage (2001–2014) and third stage (2015–2020). The years 2001 and 2014 were the key turning points. Noteworthily, considering that Tai Chi originated in ancient China and was not included in English articles in the early years, the data of WoSCC may only reveal the Western world’s understanding of traditional Chinese Tai Chi applications, which did not represent the whole development of Tai Chi. In 1981, an Australian scholar named Koh [8] published a work entitled “Tai Chi Chuan” and introduced the development history of Tai Chi and its movement description. Fifteen years later in 1996, Wolf, et al. [48] did an investigation to compare the effect of Tai Chi and computerized balance training in reducing frailty and falls in old populations, and concluded that a moderate Tai Chi intervention can impact favorably on defined biomedical and psychosocial indices of frailty. This discovery attracted widespread attention at that time and it still had enlightenment and reference significance in recent years. After 2000, a great number of scholars have contributed to the popularization of Tai Chi in the new century. However, their studies were not widely recognized and promoted. In 2004, Wang et al., [49] reviewed the effectiveness of Tai Chi on health benefits in elder patients with several chronic conditions, and found that Tai Chi can not only improve physiological and psychosocial performances, but also promote balance control, flexibility, and cardiovascular fitness in a safe and effective way. In 2012, Li et al. [50], focused on the therapeutic effect of Tai Chi on Parkinson’s patients and revealed that Tai chi training appeared to reduce balance impairments in patients with mild-to-moderate Parkinson’s disease, which broadened the globe’s horizons of the application of Tai Chi in supplementary medicine and rehabilitation. These publications significantly contributed to the advancement of Tai Chi research.

Information about regional distributions in Tai Chi research were shown in Table 1 and Figure 2. Among the top 10 most prolific countries, seven were developed countries, and only three countries were still in the developing stage. We can observe that Tai Chi studies were mainly distributed in Asia, North America and Europe, besides that, the three countries in East Asia, China, Japan and South Korea, were leading the way. China, the most prolific country, published 503 studies over the past four decades. Developed countries like the United States, Australia, Canada, England also invested a great amount of resources in the Tai Chi field. Considering that people living in developed regions usually have a longer lifespan and seem to pay more attention to fitness and wellness, so they behave with considerable enthusiasm for different ways to keep healthy and recover from diseases. In institution analysis, seven of the top 10 institutions were colleges and universities, which implied that colleges and universities were the main forms of research groups. Harvard University ranked first both in publication and centrality, demonstrating its dominant position in this field. However, seven of the top 10 institutions were from China. Considering that China is the birthplace of Tai Chi, there were more scholars in China investigating and promoting Tai Chi to the world.

As shown in Figure 3A, we can find several journals and topics which were involved in Tai Chi research by observing the network map of journals and cluster view map of reference co-citation analysis. The journals of complementary medicine, gerontology and rehabilitation therapy seemed to be the mainstream. Sports science and public health-related journals were also actively interested in this field. In addition, some comprehensive journals also played an important role in publishing Tai Chi articles. In a nutshell, the amounts and categories of journals that published Tai Chi research presented a trend of blossoming.

Some remarkable authors were discovered in this study. Authors from diverse countries and institutions who participated in the Tai Chi research all made huge contributions in this field. From Figure 3B,C, we can find out that the relationship between co-cited authors seemed stronger, while the authors cooperated only in several independent sections and lacked communication in general. Author and reference information may provide clues to dig into potential partnerships. For example, Li and Hong, et al., [2] in 2000 conducted a review to assess the characteristic effects of Tai Chi on metabolism and cardiorespiratory indexes, disclosing that Tai Chi was beneficial to cardiorespiratory function, immune capacity, mental control, flexibility, and balance control. Wang, et al., [21] in 2010 reviewed the influence of Tai Chi on psychology through meta-analysis, proving that Tai Chi was associated with positive effects in psychological wellness containing reduced stress, anxiety, depression and mood disturbance, and increased self-esteem. Wayne, et al., [51] in 2014 explored and critically evaluated the influence of Tai Chi on cognitive functions, reporting that Tai Chi may be an potential method to improve cognition of aged groups, especially strengthened the realm of executive function in healthy individuals. Chen, et al., [52] in 2016 summarized the effectiveness of Tai Chi in individuals with four common chronic conditions—cancer, osteoarthritis, heart failure and chronic obstructive pulmonary disease, and concluded that Tai Chi could be considered as an complementary therapy in the rehabilitation of these chronic diseases.

On the basis of the analysis of countries, institutions, authors, references and keywords, we summarized that the following concerns “intervention method”, “targeted therapy”, “applicable population”, “risk factors”, and “research quality” were current hotspots and expected to occur frequently over the near future and guide development framework. Here were five noteworthy topics in Tai Chi’s current concerns:

(i) Intervention method: As a rehabilitation approach, Tai Chi therapy has been deeply studied and broadly promoted. As the proverb goes: “there is more than one way to skin the cat”, and different forms of Tai Chi are practiced and promoted. While the diverse treatment type, duration and frequency of Tai Chi practices may induce different effects, so the “dose” of “Tai Chi medicine” to produce effect is still questionable and worthy of further research.
(ii) Targeted therapy: As an exercise prescription, Tai Chi may have targeted and potential therapeutic effects for several diseases. Considering that Tai Chi is a mind-body exercise accompanied by slow and graceful movements, Tai Chi appears to be associated with improvements in motor [53,54,55], behavioral [21,56,57], cardiorespiratory [18,58,59] and cognitive [2,60,61] functions. However, the cross-talk mechanism of these effects is still unclear, besides that, more regulatory factors and circulatory pathways are worthy of investigating to prove the adaptation induced by Tai Chi.
(iii) Applicable population: Tai Chi is an all-age applicable strategy while most participants practicing it are middle-aged and elderly people. Aging is a leading issue nowadays. As the aging process intensifies, degenerative disorders include osteoarthritis, stoke, Parkinson’s and other diseases may distributed both the patients and their families [52]. More studies aimed at specific populations, such as people who are prone to falls, breast cancer sufferers, and patients with cardiovascular, cerebrovascular and brain related diseases should be individually designed and carried out to clarify the effect and quality of Tai Chi intervention.
(iv) Risk factors: Several factors (for instance, sample size, age, duration of intervention, countries and regions, whether there are complications) affect the results of Tai Chi research, leading to inconsistent conclusions and recommendations. Different conclusions may occur in correspondence with variations in designs, comparisons, heterogeneous outcomes and inadequate controls. In the next stage, scholars should attempt to reduce the risk bias caused by the research objects and experimental designs to improve the universality and generalization of the study.
(v) Research quality: Risk factors are triggers for the uneven quality of research. Therefore, extensive systematic reviews and meta-analysis have sprung up in recent years to evaluate the ultimate intervention effects of Tai Chi. More high-quality, well-controlled trials with longer follow-up periods are urgently needed to draw definitive conclusions and better guide clinical decisions.

From the reference knowledge map and keywords distribution presented in Figure 4 and Figure 5, we can also observe that the topics on Tai Chi field over the past four decades were generally in the form of: (i) effects of Tai Chi on physical performance and executive function related to balance, posture control, gait, and fall prevention; (ii) multiple physiological and psychological mechanisms of Tai Chi on body metabolism; (iii) research objects and target population of Tai Chi; and (iv) development tendency of Tai Chi and its application in chronic disease prevention and rehabilitation. By analyzing these domains, we can understand the effects of Tai Chi in a more comprehensive manner. In addition, several burst keywords reflected the applications of Tai Chi in the health promotion field. From Figure 5C, we concluded five prevalent domains that Tai Chi can be applied to in health science. Analyses of these five domains follows:

(i) Fall risk: Falls can induce serious consequences, such as fractures and brain injuries [62,63] and Tai Chi is a popular choice for fall prevention and balance training [64,65]. By practicing Tai Chi, the proprioception loss and motor disturbances caused by aging may improve in part and then postural imbalance and fall risks would be decreased. Wolf and his colleagues [48] found that three potential risk factors—having fallen within the past year, fear of falling, and ease of falling asleep at night—had a statistically significant reduction after a 15 weeks moderate Tai Chi intervention. Actually, by comparing three different interventions including Tai Chi, computerized balance training, and an education exercise-control condition, Wolf et al., also suggested that except for favorable effects upon the occurrence of falls, Tai Chi can impact positively on defined biomedical and psychosocial indices of frailty as well. A previous meta-analysis based study [66] also proved this results and concluded that Tai Chi, as a balance training method, has its unique advantages in substantially reducing fall rates. Most recently, an abridged Cochrane systematic review [67] with 2655 samples included verified that Tai Chi or other similar exercise interventions may reduce the rate of falls by 19% compared with control groups.
(ii) Cardiorespiratory related diseases: Common cardiorespiratory diseases include coronary heart disease, myocardial infarction, chronic obstructive pulmonary disease, asthma, etc., which have brought increasing health burdens worldwide. Tai Chi exercise is a clinically proven and cost-effective intervention that can prevent or cure chronic cardiorespiratory disorders. As an endurance training method, Tai Chi could reverse, at least in part, the age-related decline in aerobic power. Lai and Hong et al. [68,69] evaluated the effects of 24 months of regular Tai Chi training on the maintenance of cardiovascular function in elder groups and found a smaller decline in the maximal oxygen uptake compared with sedentary counterparts. By the application of breathing regulation in continuous Tai Chi exercise, the metabolic and cardiorespiratory functions of participators might gradually be improved [2,49].
(iii) Stroke: Stroke survival is often accompanied by executive function degradation and other non-motor dysfunction, such as mental or sleep disorder and cognitive impairment. A growing body of evidence [70,71,72] has suggested that Tai Chi is an effective treatment for post-stroke patients. Due to the advantages of soft, slow and low-intensity movements, Tai Chi can be considered as a safe and relieved strategy for stroke patients in rehabilitation process. The Tai Chi exercise concept of “taking the waist as axis” advocates the active contraction of the trunk of patients. During the practicing procedure, the lower limbs of stroke patients are often in a semi flexed state (eccentric isotonic contraction of quadriceps femoris), which is conducive to strengthening the proprioceptor around the knee joint [73]. For the upper limbs, “cloud hand” and other movements can promote the recovery of proprioception, improve the motor function and coordination of the upper limbs [74,75], which is conducive to the pain management of stroke patients, and treatment of the shoulder hand syndrome after stroke.
(iv) Parkinson’s disease: Parkinsonism is a common disease among the elderly. It is defined as motor abnormalities and non-motor manifestations [76,77]. Previous research has showed the positive evidence that Tai Chi had beneficial effects in improving motor function, balance and functional mobility in people with PD [50,76,78]. When practicing Tai Chi, the strength of the patients’ lower limbs and stability could be improved with the multiple direction movements and mind concentration. Besides, Tai Chi includes many spiral diagonal movements, which can improve the awakening of the central nervous system and coordination of upper and lower limbs. Apart from motor function, a previous article [79] demonstrated Tai Chi positively impacts multiple non-motor symptoms, especially in the quality of life. However, Amano et al., [80] found that 16 weeks of class-based Tai Chi did not significantly improve either gait initiation, gait performance, or reducing Parkinsonian disability in the subset of Parkinson’s disease patients. Thus the use of short-term Tai Chi exercise is understudied. In a word, Tai Chi exercise can improve the initiative and enthusiasm of Parkinson’s patients to participate in rehabilitation training actively, while the dose to induce these positive changes is needed to be further investigated.
(v) Depression: As one of the most common psychiatric conditions worldwide [81,82,83], depression can affect both physical and psychological health in all-aged groups. Multiple research has reported that non-pharmacological therapies like Tai Chi might be useful to alleviate depression symptoms [20,84,85]. Tai Chi can not only strengthen the body, but also improve the mood and help depressive patients build confidence and relieve anxiety. Additionally, regular Tai Chi activities with groups can help people shape healthy living habits, due to the group and social interaction, the health benefits may be further amplified. Loneliness is a general concern among the elderly, which is one of the trigger points of developing depression [86,87] and regular group exercise like Tai Chi may help to counteract this.

Noticeably, in the context of the coronavirus disease 2019 (COVID-19) pandemic [88,89], the isolation may lead to less exercise and more health-related problems [90,91]. As COVID-19 was spreading fast starting late 2019, the world faced unprecedented medical challenges. Many of the infected patients suffered from severe respiratory impairment, accompanied with cardiac disability [92,93]. Considering that elder groups’ cardiopulmonary and immune functions are on the decline phase, they are prone to be infected with COVID-19. However, Tai Chi has its unique advantage as a home-based exercise that can be practiced and performed to improve heart function, blood circulation, respiratory function, and so on. Recently, one study has preliminarily proved that alternative therapy like Tai Chi made sense in improving the quality of life, anxiety, depression, distress, and fatigue of cancer patients during COVID-19 period [94]. In addition, more ongoing studies are now implemented to verify the effectiveness and safety of Tai Chi for COVID-19 in rehabilitation period [95,96]. We are confident that Tai Chi and other exercise therapies can play an important role as a supplement to clinical medicine in the treatment of COVID-19 and other health promotion fields.

There are multiple strengths in the current study. Unlike previous meta-analysis focusing on effects of Tai Chi in several narrow perspectives, this study had a bird’s eye view of the Tai Chi’s current hotspots and research frontiers. In addition, it is one of the pioneering studies to identify countries, institutions, journals, authors, references that have played important roles in global Tai Chi research. The conclusions of our research provide suggestions of promising domains that Tai Chi can be applied to and point out existing concerns in the recent literature. Moreover, the study is relevant to the journal’s scope and has a wide audience. With detailed exploration of the literature with vivid figures and appropriate tables, we hope this study would be beneficial to all readers of related scholars, doctors, sports therapists and ordinary Tai Chi enthusiasts, etc.

Some limitations of this study due to methodological problems should be noted. Firstly, the data collection was only limited to the Web of Science Core Collection (WoSCC) database, which may lead to publication bias to some extent. However, the WoSCC database has recognized the quality of its papers, and this database has been widely applied for the retrieval of nature science researches and selected as the data source of multiple high-quality bibliometric studies. Additionally, to better present results and guarantee the quality of all included literature, only articles and reviews published in English were included. This resulted a screening bias and thus our review might not fully represent all studies in the research field. Thus, these limitations need to be considered when studying the findings of this investigation.

## 5. Conclusions

This study applied a bibliometric evaluation to provide valuable details for global Tai Chi research progress and hotspots. A total of 1078 records were retrieved from the WoSCC database and the trend of annual publications displayed notable growth overall, especially in the most recent five years. The results part presents the main countries, institutions, journals, authors and references that contributed to the Tai Chi domain (Q1), which were investigated in-depth via an analysis of the thematic network structures of each theme found. We considered that the frontiers and promising domains of Tai Chi associated with health promotion are “Fall risk”, “Cardiorespiratory related disease”, “Stroke”, “Parkinson’s disease”, and “Depression” (Q2). In addition, “Intervention method”, “Targeted therapy”, “Applicable population”, “Risk factors”, and “Research quality” are current concerns and difficulties in Tai Chi research (Q3). As an exercise prescription and health promotion approach, Tai Chi has received increasing attention, and more high-quality randomized controlled trials are urgently needed to draw definitive conclusions and guide clinical decisions.

## Figures and Tables

**Table 1:** Ranking of top 10 countries and institutions in the field of Tai Chi research from 1980 to 2020.

Rank	Country	Publications	Centrality	Institution	Publications	Centrality
1	China	503	0.38	Harvard Univ	74	0.23
2	United States	394	0.66	Chinese Univ Hong Kong	51	0.08
3	Australia	67	0.09	Hong Kong Polytech Univ	45	0.06
4	Canada	59	0.08	Shanghai Univ Sport	39	0.09
5	England	49	0.04	Brigham & Womens Hosp	27	0.01
6	South Korea	47	0.03	Chinese Acad Sci	25	0.03
7	Japan	27	0.01	Oregon Res Inst	23	0.03
8	Germany	17	0.01	Beth Israel Deaconess Med Ctr	21	0.01
9	Italy *	14	0.01	Fujian Univ Tradit Chinese Med	20	0.01
10	Poland *	14	0.01	Univ Arizona *	19	0.01
				Chang Gung Univ *	19	0.02

The countries and institutions listed in the table are ranked independently; * indicates a tie for equal place.

**Table 2:** Ranking of top 10 journals and co-cited journals in the field of Tai Chi research from 1980 to 2020.

Rank	Journal	Publications	Percentage (%)	IF (2019)	Cited Journal	Co-Citation Counts
1	*Evidence Based Complementary and Alternative Medicine*	42	3.90%	1.813	*Journal of the American Geriatrics Society*	599
2	*Archives of Physical Medicine and Rehabilitation*	31	2.88%	3.098	*Archives of Physical Medicine and Rehabilitation*	534
3	*Complementary Therapies in Medicine*	25	2.32%	2.063	*Medicine and Science in Sports and Exercise*	424
4	*American Journal of Chinese Medicine*	22	2.04%	3.682	*British Journal of Sports Medicine*	361
5	*Journal of the American Geriatrics Society*	20	1.86%	4.180	*Journal of Alternative and Complementary Medicine*	342
6	*Medicine*	19	1.76%	1.552	*Journals of Gerontology Series A-biological Sciences and Medical Sciences*	306
7	*Journal of Aging and Physical Activity*	18	1.67%	1.763	*Physical Therapy*	286
8	*Plos One*	16	1.48%	2.740	*Archives of Internal Medicine*	272
9	*Research in Sports Medicine*	15	1.39%	2.554	*American Journal of Chinese Medicine*	268
10	*Journal of Sport and Health Science*	14	1.30%	5.200	*New England Journal of Medicine*	258

**Table 3:** Ranking of top 10 authors, co-cited authors, and co-cited references in the field of Tai Chi research from 1980 to 2020.

Rank	Author	Counts	Co-Cited Author	Counts	Co-Cited Reference	Counts
1	Wayne PM	32	Li FZ	351	Li FZ, 2012, NEW ENGL J MED, V366, P511	65
2	Liye Zou	17	Lan C	333	Wayne PM, 2014, J AM GERIATR SOC, V62, P25	47
3	Lidian Chen	16	Wang CC	308	Wang CC, 2004, ARCH INTERN MED, V164, P493	39
4	Li FZ	15	Wolf SI	308	Wang CC, 2010, BMC COMPLEM ALTERN M, V10	36
5	Jing Tao	14	Wayne PM	243	Wang F, 2014, INT J BEHAV MED, V21, P605	35
6	Taylor-piliae Re	13	Wu G	176	Yeh GY, 2011, ARCH INTERN MED, V171, P750	35
7	William W N Tsang	13	Yeh GY	167	Wang CC, 2009, ARTHRIT RHEUM-ARTHR, V61, P1545	31
8	Guohua Zheng	12 *	Li JX	164	Li FZ, 2005, J GERONTOL A-BIOL, V60, P187	31
9	Yeh GY	12 *	Taylor-piliae Re	149	Wang CC, 2010, NEW ENGL J MED, V363, P743	30
10	SI Wolf	12 *	Lee MS	147	Hong YL, 2000, BRIT J SPORT MED, V34, P29	29 **
11	Lan C	12 *			Taylor-Piliae RE, 2014, ARCH PHYS MED REHAB, V95, P816	29 **

* indicates a tie for 8th place, ** indicates a tie for 10th place.

## Data Availability

The data that support the findings of this study are available from the corresponding author, upon reasonable request.
